# Measurement of a 3D Ultrasonic Wavefield Using Pulsed Laser Holographic Microscopy for Ultrasonic Nondestructive Evaluation

**DOI:** 10.3390/s18020573

**Published:** 2018-02-13

**Authors:** Xing Wang, Guang-Ming Zhang, Hongwei Ma, Yishu Zhang, Doudou Wang

**Affiliations:** 1 School of Mechanical Engineering, Xi’an University of Science and Technology, Xi’an 710054, China; X.Wang@2017.ljmu.ac.uk (X.W.); mahw@xust.edu.cn (H.M.); Y.Wang1@2017.ljmu.ac.uk (Y.Z.); 2 General Engineering Research Institute, Liverpool John Moores University, Liverpool L3 3AF, UK; g.zang@ljmu.ac.uk; 3 School of Science, Xi’an University of Science and Technology, Xi’an 710054, China; wangdoudou@xust.edu.cn

**Keywords:** digital holographic microscopy, CCD sensor, array transducer, ultrasonic wavefield, ultrasonic imaging

## Abstract

In ultrasonic array imaging, 3D ultrasonic wavefields are normally recorded by an ultrasonic piezo array transducer. Its performance is limited by the configuration and size of the array transducer. In this paper, a method based on digital holographic interferometry is proposed to record the 3D ultrasonic wavefields instead of the array transducer, and the measurement system consisting of a pulsed laser, ultrasonic excitation, and synchronization and control circuit is designed. A consecutive sequence of holograms of ultrasonic wavefields are recorded by the system. The interferograms are calculated from the recorded holograms at different time sequence. The amplitudes and phases of the transient ultrasonic wavefields are recovered from the interferograms by phase unwrapping. The consecutive sequence of transient ultrasonic wavefields are stacked together to generate 3D ultrasonic wavefields. Simulation and experiments are carried out to verify the proposed technique, and preliminary results are presented.

## 1. Introduction

In many industries such as automotive, aerospace, shipping and railway, ultrasonic imaging is widely used for the engineers to intuitively find the defects inside workpieces [1]. An ultrasonic piezo array transducer is normally used in ultrasonic array imaging, for example phase array C-scan [2,3]. An array transducer is composed of multiple independent piezoelectric elements that are excited according to certain rules and timing in order to adjust the focal position and steer the ultrasonic beam direction [4]. The performance of phased array technology is affected by the size and configuration of piezoelectric elements [5]. In this paper, a CCD (Charge-coupled Device) sensor acting as the ultrasonic receiving array overcomes many challenging issues faced by the current ultrasonic transducer arrays, such as element density and element spacing and aperture, increasing the imaging performance. As it is a non-contact sensing technique in the receiving phase then this could circumvent problems when the surface is rough or has a complex geometry.

The optical detection techniques for ultrasound are classified into non-interferometric techniques and interferometric techniques. The former are well developed or of limited application, while the latter are more general and are presently the object of active developments [6]. The noninterferometric techniques, such as knife-edge technique, is very insensitive to vibrations but requires a good surface finish and is hardly applicable to image the 3D ultrasonic wavefield [7].

In interferometric techniques, a Michelson interferometer and other configurations based on the other two wave interferometers (Mach-Zehnder, Fiseau) are normally used to receive the ultrasound [8]. Hochreiner et al. report on the remote three-dimensional photoacoustic imaging by utilizing a two-wave mixing interferometer (TWMI) and the Fourier domain synthetic aperture focusing technique (FSAFT) [9]. The TWMI setup can detect rough and flat surfaces, but the imaging of the sample was done by raster scanning in x- and y-direction. Therefore, the technique cannot strictly detect the ultrasonic wavefield with full-field and there is no advantage in the measurement of high-frequency ultrasonic wavefields.

In recent years, the electronic speckle interference techniques have been proposed for use in non-contact detection of ultrasonic signals [10]. Mast et al. report that the two-dimensional ultrasonic surface wave data are obtained by optical electronic speckle pattern interferometry (ESPI) techniques [11]. The speckle interference with digital phase-stepping is used to capture traveling ultrasonic Lamb waves [12]. This means either that the phase to be measured should be constant over the time required for acquisition of phase-shifted interferograms or that compensation needs to be introduced to allow a phase value to be calculated at the time of each recorded frame rather than once every four frames [13]. Ishikawa et al. investigated the use of parallel phase-shifting interferometry (PPSI) with a high-speed polarization camera for imaging a sound field in air [14]. Although the phase-shifted images are captured by a single-shot using PPSI, the sound field in the opaque solid specimen was not obtained. These limitations can be a disadvantage when high-frequency ultrasound need to be investigated in non-destructive testing applications.

However digital holography can measure phase and amplitude information directly with one hologram. Holography is a technique for recording and reconstructing static or dynamic wavefronts. Holographic interferometry allows the comparison of wavefronts recorded at different time instants [15] and has been used for vibration measurement since 1965 [16]. Other applications of this technique include displacement analysis of solid objects, shape measurement, and investigation of the refractive-index change in transparent media [17]. Pedrini et al. described a method for measuring dynamic events in which digital holograms of an object are recorded on a high-speed CCD [18]. The phases of the wavefront recorded at different times are calculated, only one image hologram is needed for the phase to be determined at a given time instant [19]. Matoba et al. propose an optical voice recorder based on digital holography for recording and reproducing propagating sound waves in air [20].

In this paper, a method to record 3D ultrasonic wavefields on the basis of digital holographic interferometry is proposed for ultrasonic non-destructive evaluation.

## 2. Measurement of 3D Ultrasonic Wavefields Using Digital Holographic Interferometry

### 2.1. Theory of Digital Holographic Interferometry

The amplitudes of the ultrasonic wavefield are a few nanometers to a few microns. When the ultrasonic wavefield is measured, the speckle field will cover the amplitudes. The amplitudes and phases of the ultrasonic wavefield are difficult obtain with the digital hologram technique due to the tiny amplitudes. However, digital holographic interferometry can be used to measure phase change from speckle field [21]. According to holographic interferometry theory [22], the first hologram is collected when the surface of test piece is stationary, and the second hologram is collected when the test piece is slightly displaced or excited. Based on the phase information provided by the two holograms, the interferogram of the surface of test piece can be calculated. Then, the ultrasonic wavefield can be obtained from the interferogram [23].

Setting the wave intensity distribution to a constant value, the phase distribution only changes when the surface of test piece deforms. In the first exposure (*t* = *t*1), the corresponding light intensity (object light and reference light) distribution on the CCD camera is

(1) $$I_{1}\left(x,y\right)={\left|O_{1}\left(x,y\right)+R\left(x,y\right)\right|}^{2}$$

(2) $$O_{1}\left(x,y\right)=O_{0}\left(x,y\right)\exp\left[-j\Phi_{01}\left(x,y\right)\right]$$

(3) $$O_{1}\left(x,y\right)=R_{0}\exp\left[-j\Phi_{R}\left(x,y\right)\right]$$

where $O_{1}\left(x,y\right)$ represents the object light and $O_{0}\left(x,y\right)$ represents intensity of object light and $\Phi_{01}\left(x,y\right)$ represents the phase distribution of object light. R(x,y) represents the reference light and $R_{0}$ represents intensity of object light, $\Phi_{R}\left(x,y\right)$ represents the phase distribution of object light. Setting the exposure time of the hologram (*t*1) as $T_{1}$, the photometric exposure is

(4) $$H_{1}=I_{1}T_{1}={\left|O_{1}\left(x,y\right)+R\left(x,y\right)\right|}^{2}T_{1}$$

In the second exposure (*t* = *t*2),$\;O_{2}\left(x,y\right)$ represents the object light at *t*2 and the intensity distribution of the object light $O_{0}\left(x,y\right)$ remains unchanged, and the phase distribution $\Phi_{01}\left(x,y\right)$ change to $\Phi_{02}\left(x,y\right)$ in the second exposure.

(5) $$O_{2}\left(x,y\right)=O_{0}\left(x,y\right)\exp\left[-j\Phi_{02}\left(x,y\right)\right]$$

If the reference light R(x,y) remains unchanged, the light intensity distribution on the CCD is

(6) $$I_{2}\left(x,y\right)={\left|O_{2}\left(x,y\right)+R\left(x,y\right)\right|}^{2}$$

where $O_{2}\left(x,y\right)$ is the object light in the hologram (*t*2). Setting the exposure time of the hologram (*t*2) to $T_{2}$, the corresponding photometric exposure is

(7) $$H_{2}=I_{2}T_{2}={\left|O_{2}\left(x,y\right)+R\left(x,y\right)\right|}^{2}T_{2}$$

The total exposure volume is

(8) $$E=H_{1}+H_{2}={\left|O_{1}\left(x,y\right)+R\left(x,y\right)\right|}^{2}T_{1}+{\left|O_{2}\left(x,y\right)+R\left(x,y\right)\right|}^{2}T_{2}$$

When reconstructing *E* with reference light R(x,y) in Equation (3), the ‘+1’ diffracted light can be described as

(9) $$I_{3}=\beta \left(T_{1}O_{1}+T_{2}O_{2}\right)RR^{*}={\left(\;\beta \left|O_{1}O_{2}\right|\right)}^{2}\left\{T_{1}^{2}+T_{2}^{2}+2T_{1}T_{2}cos\Delta \Phi \left(x,y\right)\right\}$$

(10) $$\Delta \Phi \left(x,y\right)=\Phi_{02}\left(x,y\right)-\Phi_{01}\left(x,y\right)$$

ΔΦ(x,y) is the change in phase distribution between $O_{2}\left(x,y\right)$ and $O_{1}\left(x,y\right)$. 𝛽 is a real constant.

By setting

(11) $$W_{3}={\left(\;\beta {\left|R\right|}^{2}\left|O_{1}O_{2}\right|\right)}^{2}\left(T_{1}^{2}+T_{2}^{2}\right)$$

(12) $$V_{3}=\frac{2T_{1}T_{2}}{T_{1}^{2}+T_{2}^{2}}$$

We have

(13) $$I_{3}=W_{3}\left\{1+V_{3}cos\Delta \Phi \left(x,y\right)\right\}$$

where $V_{3}=1$ corresponds to the optimum fringe contrast. To get the optimum fringe contrast, according to Equation (9), make the two exposure times equal: $T_{1}=T_{2}$ [23]. The interferogram of ultrasonic wavefield at *t*2 moment can be described as

(14) $$I_{3}=2W_{3}\cos^{2}\left[\frac{\Delta \Phi \left(x,y\right)}{2}\right]$$

According to Equation (14), the information of the ultrasonic wavefield at *t*2 is embedded in the interferogram. The ultrasonic wavefield at *t*2 moments can be recovered from the interferogram through phase unwrapping (details can be found in Section 4).

### 2.2. Measurement of 3D Ultrasonic Wavefields

**Figure 1:**
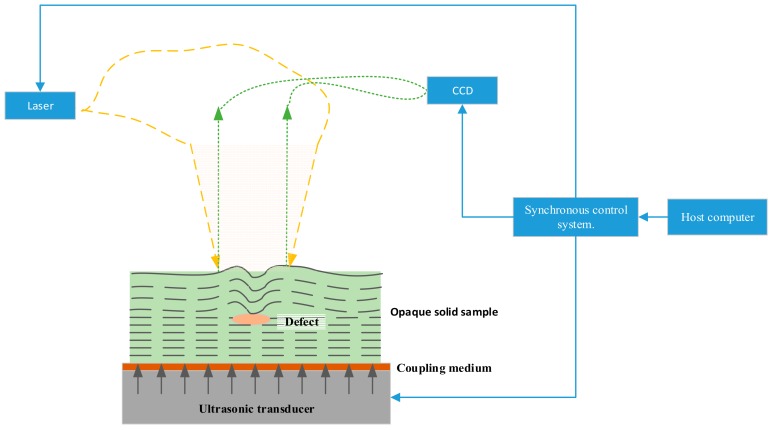
Schematic diagram of the proposed measurement method (CCD: Charge-coupled Device).

In this paper, a method based on digital holographic interferometry is proposed to record 3D ultrasonic wavefields instead of the array transducer for imaging the internal defects of test piece. As shown in Figure 1, this technique works as follows: an opaque solid sample is put on top of a piezo that generates a single frequency short-pulse ultrasonic wave, and the ultrasonic wave propagates to the sample surface. These ultrasonic wavefields carry information about the internal structures and the internal defects. Then the dynamic ultrasonic wavefield on the surface is measured by a lensless CCD camera. 3D ultrasonic data are captured by recording multiple ultrasonic wavefields at a consecutive time sequence by synchronizing the CCD capture, pulsed laser irradiation and ultrasonic transducer excitation.

**Figure 2:**
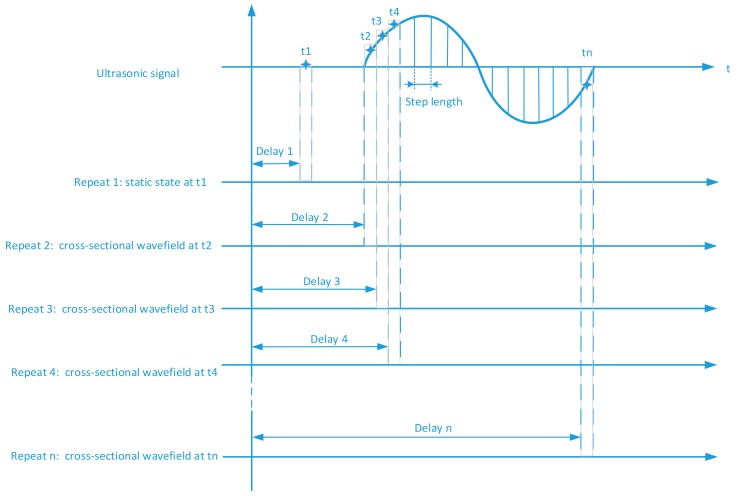
Acquisition of 3D ultrasonic wavefields consisting of multiple cross-sectional wavefields.

By shifting the delay time between the ultrasonic excitation and CCD camera capture, and repeating the optical measurement, the cross-sectional wavefields at different depths of the test sample can be recorded. The time sequence of ultrasonic wavefields form two 3D arrays (two spatial dimensions + depth): a phase array and an amplitude array. The spatial dimensions of 3D ultrasonic wavefields are determined by the number of elements on the CCD camera. Each element of the CCD camera captures an equivalent ultrasonic A-scan signal. The sampling frequency for acquiring these ultrasonic A-scan signals is determined by the step length of the time delay shifting shown in Figure 2. The minimum step length is the length of the laser pulse. If the step length is less than the laser pulse length, two measured wavefields will be overlapped, and thus will reduce the accuracy of the wavefield measurement. Therefore, the laser pulse width determines the upper limit of sampling frequency for the A-scan signal acquisition using the optical measurement. In addition, the repetition rate of the pulsed laser and the frame rate (fps) of the high-speed camera determine the 3D ultrasonic wavefield acquisition speed.

As shown in Figure 2, *t*1–*tn* are the different time points in an ultrasonic signal. The Delay 1 in Figure 2 is the delay time at first measurement (Repeat 1). At *t*1, the sample has not yet been excited by the ultrasonic signal, and the corresponding hologram is to measure the topographical surface of the test piece in the static state. The sampling points of the dynamic ultrasonic wavefields are *t*2–*tn*. By shifting the delay time (Delay 2) in second measurement (Repeat 2), the cross-section wavefield at *t*2 is obtained. Delay 3–Delay n are the delay time at t3– *tn* moment and the different delay times are based on the cross-sectional wavefields at different depths. As shown in Figure 2, each repeated measurement will set a delay time and the corresponding ultrasonic wavefields will be obtained.

**Figure 3:**
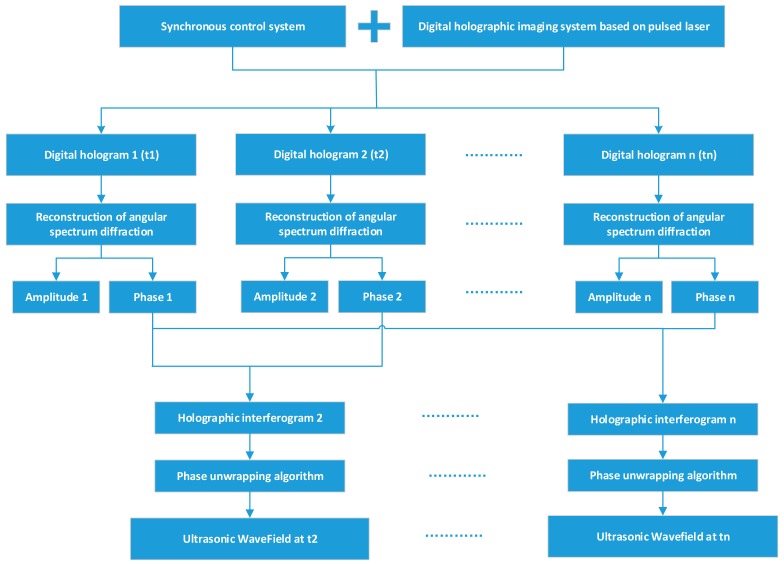
Flow chart of measurement of 3D ultrasonic wavefields.

Figure 3 shows an analysis of *n* points. As shown in Figure 3, the digital holograms of the test sample are obtained at times t1, t2– tn. The specimen surface at t1 is stationary. Amplitude 1 and phase 1 are related to Equation (2). Amplitude 2 and phase 2 are related to Equation (5). According to the theory of digital holographic interferometry (in Section 2.1), the interferograms 2 to *n* − 1 of the ultrasonic wavefields at *t*2 to tn relative to t1 can be obtained. Holographic interferogram2 in Figure 3 is linked to Equation (9). As shown in Figure 3, the angular spectrum reconstruction is used to obtain the amplitude and phase of the light field. The angular spectrum method has several advantages over the more commonly used Fresnel transformation or Huygens convolution method. Spurious noise and interference components can be tightly controlled and the reconstruction distance does not have a lower limit. The off-axis angle between the object and reference can be lower than the Fresnel requirement and still be able to cleanly separate out the zero-order background [24]. These interferograms are then used to generate the cross-sectional wavefields at time instants of *t*2 to *tn*.

## 3. Design of 3D Ultrasonic Wavefields Measurement System

**Figure 4:**
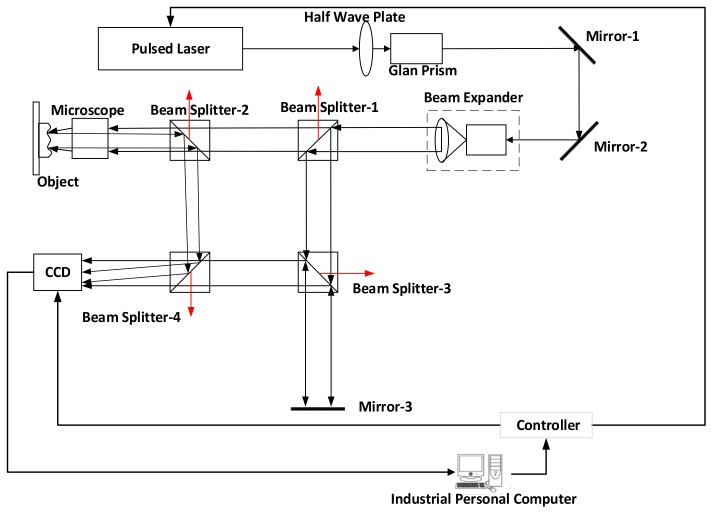
The designed pulsed digital holographic microscopy system.

In order to obtain the interferograms in Figure 3, a pulsed digital holographic microscopy system is designed as shown in Figure 4.

First, the pulsed light beam passes through the half-wave plate and Glan prism. They can both control the energy of the light beam and ensure a pure polarized light beam, improving the efficiency of the pulsed light beam interference. Then, the light beam passes through the beam expander and enters beam splitter-1. The coherence distance of the pulsed laser is limited. In order to ensure that the light path of the reference light and the object light are the same, the Mirror-3 in Figure 4 is used to make up the light path difference. The specimen is an opaque solid and the reflected object light is weak when the reflectivity of the target specimen is low. To facilitate the ratio of intensity between the reference beam and object beam, we chose a 9:1 non-polarizing beam splitter cube (NPBS) as beam splitter-1. The NPBS used here is 90% of the transmitted light and 10% of the reflected light, which enhances the intensity of the object light and weakens the intensity of the reference light. It is also helpful to tune the ratio of the object light to the reference light, improving the hologram quality. Because the pulsed light beam is pure polarized light, we chose the rest of the beam splitters to be NPBS, but with a splitter ratio of 5:5. As shown in Figure 4, the object is placed near beam splitter-2, and mirror-3 is placed near diagonal beam splitter-3. The four beam splitters in Figure 4 reflect the unwanted light beam out of the light path, as shown by the red arrow.

The parameters of the pulsed laser, camera and microscopic lens are crucial for acquiring high-quality 3D ultrasonic wavefields. The laser power should provide sufficient light flux to illuminate the sample surface, satisfying the flux demand of the CCD camera. Coherence length of the laser pulse is also important. A large coherence length will greatly facilitate the construction of the digital holographic microscopy subsystem. Important parameters for the high-speed camera include the shutter speed, CCD camera sensitivity and signal to noise ratio (SNR) and the frame rate. Short shutter time, high sensitivity and SNR of the CCD camera ensure capturing a high-quality optical hologram even when limited light is provided by a short laser pulse. The performance of the microscopic lens not only reduces the optical aberration, but also the magnification and numerical aperture will affect the lateral resolution of the instrument. The larger the magnification, the smaller the imaging region, but with a higher lateral resolution. In this paper, the pulsed laser chosen here is Beamtech NIMMA 400 Pulsed Laser (Beijing, China) with a pulse width of 8 ns and the repetition rate of 1–10 Hz. The wavelength of 532 nm is used in the experiments. If the detected frequency of ultrasonic wavefield is 1 MHz, and the time period of 1000 ns, the 8 ns pulse width of the laser can illuminate one cycle of 1/125 for its 1000 ns cycle. In other words, 8 ns << 1000 ns, can be regarded as relatively transient. The pco.1600 CCD (Kelheim, German) with the shortest exposure time is chosen, and its exposure time can be as short as 500 ns. The frame rate of CCD is 30 fps. The microscopic lens used in this paper is Japan Mitutoyo company (Kawasaki, Kanagawa Prefecture, Japan), its numerical aperture NA = 0.5, the magnification of 50×.

**Figure 5:**
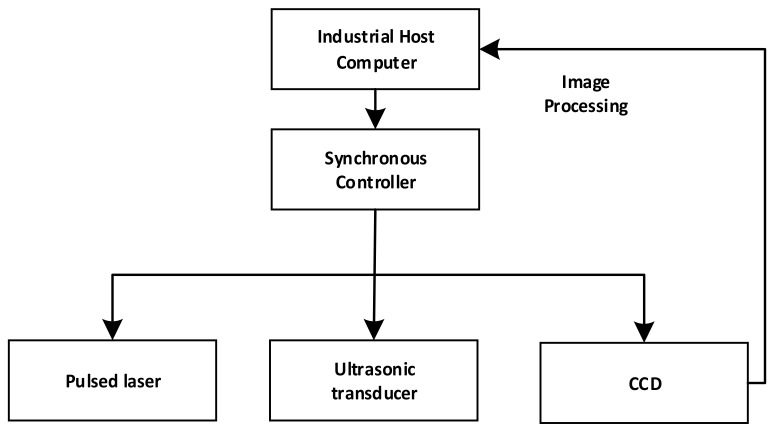
Schematic of the synchronous control system.

**Figure 6:**
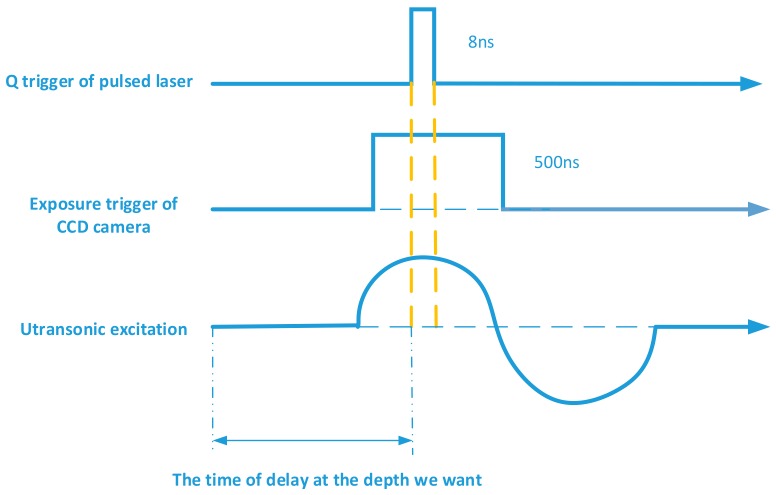
Synchronous control timing diagram (CCD: Charge-coupled Device).

Control and synchronization is fundamental for high-quality hologram capture. The control system must provide a precise delay time between the laser pulse and the camera capture for the proposed digital holographic microscopy system. Figure 5 shows the synchronous control system, consisting of a host computer, a synchronous controller, ultrasonic transducer, a CCD camera, and a pulsed laser. The synchronous controller is implemented through the timing of National Instruments PXI-6602 (London, UK) and digital I/O modules. As shown in Figure 6, the synchronous controller is designed to ensure that the CCD camera receives the 8-ns laser pulse within its exposure time window of 500 ns, and it is also the time when the cross-sectional wavefield at the depth we want.

## 4. Simulation Study

In this section, computer simulation is carried out to verify the feasibility of the proposed measurement method.

### 4.1. Simulation of Interferograms of Ultrasonic Wavefield

The interferograms of the ultrasonic wavefield are simulated by computer-generated hologram. Here, t1 is static, and a Gaussian distribution is used to simulate the deformation of the surface at the time point t2.

**Figure 7:**
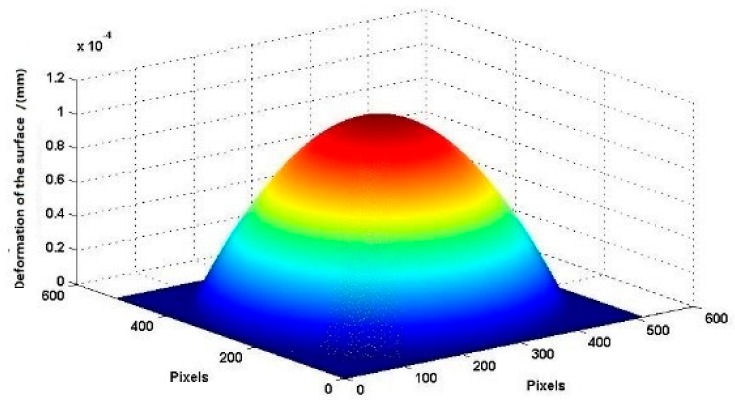
Surface of a longitudinal ultrasonic transducer.

As shown in Figure 7, the maximum deformation of the surface caused by the ultrasonic wavefield is z=115 nm at *t*2. The wavelength of the simulated light source is $\lambda =0.532\times 10^{-3}\;\mathrm{mm}$. The pixel width of the CCD is pix=7.4 µm. The angle between the reference light and the object light is π/4. The distance between the object light and the reference light has been considered in Figure 4. Therefore, the factor is not considered in the simulation section.

**Figure 8:**
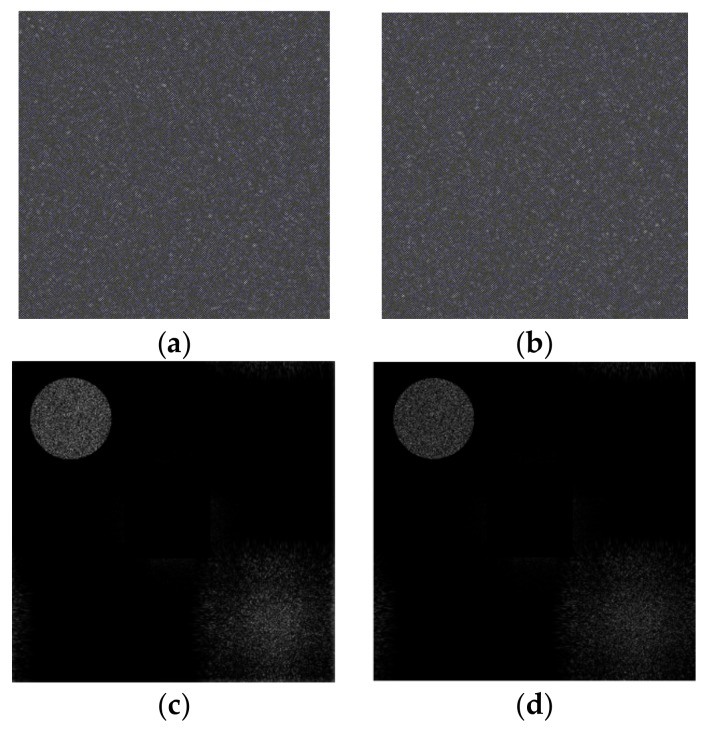
The holograms before and after deformation. (**a**) holograms (*t*1); (**b**) holograms (*t*2); (**c**) reconstructed plans (*t*1); (**d**) reconstructed plans (*t*2).

**Figure 9:**
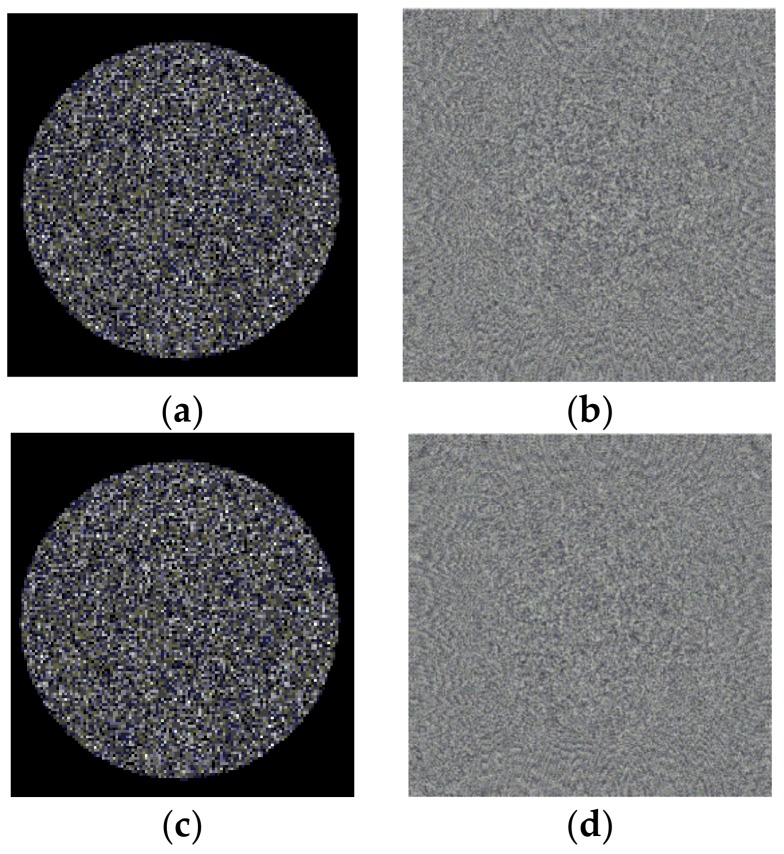
Reconstructed images before and after deformation. (**a**) Amplitude of reconstruction image (*t*1); (**b**) phase of reconstruction image (*t*1); (**c**) amplitude of reconstruction image (*t*2); (**d**) phase of reconstruction image (*t*2).

Using Fresnel diffraction theory, the object light reaching the hologram plane is simulated, and the reference light is defined. Also, the interference between the object light and reference light is simulated. The corresponding interference field intensity is calculated, and the digital holograms are formed. Using Equations (1) and (6), the holograms at *t*1 and *t*2 are obtained, as shown in Figure 8. Figure 8a is the hologram at *t*1, and Figure 8b is the hologram at *t*2. Based on these holograms, the two holograms from the angular spectrum diffraction are reconstructed. By using the angular spectrum transfer function in analytic form, the calculation required only one direct and one inverse FFT (Fast Fourier Transformation). The angular spectrum formula also rigorously satisfies the scalar wave equation, and its use is widespread in holography. Figure 8c,d shows the reconstructed plans, and the zero-order diffraction light is filtered out. After reconstructing the digital holograms, the reconstructed images of the model at *t*1 and *t*2 are obtained, as shown in Figure 9.

**Figure 10:**
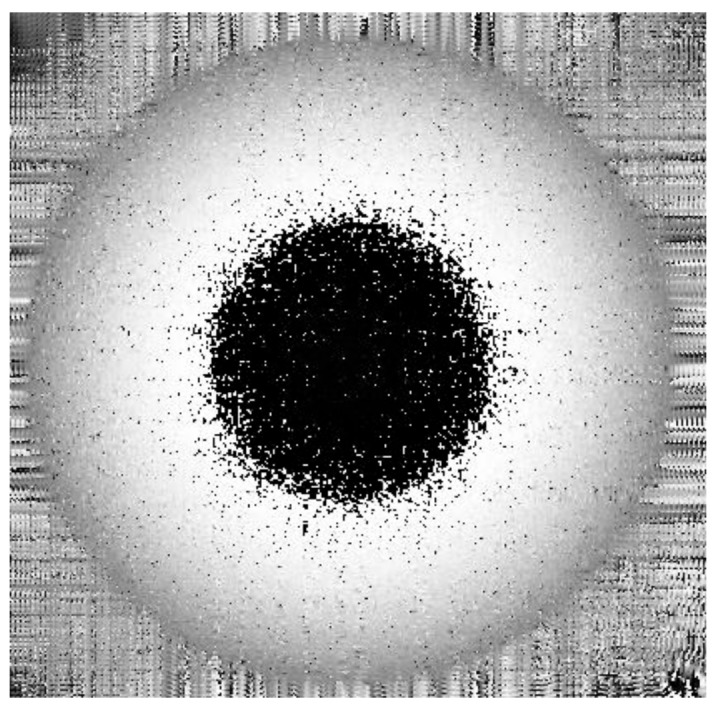
Digital interferogram of the object light field at t2 moment.

Figure 9a,c shows the amplitudes of the reconstructed image at *t*1 and *t*2, and these represent the shape of the speckled object. Figure 9a,c show that 115 nm is less than the wavelength of the illumination light. Therefore, the phase of the optical wave field is represented by the arc tangent function and varies in the range of [−π,π]. In fact, the real phase takes a value of 2π, which remains a random variable. Therefore, the phase shown in Figure 9b,d is a random distribution, and the deformation cannot be directly detected from the phase image. According to Equations (5) and (6), the digital interferogram of the object light field at *t*2 relative to *t*1 as shown in Figure 10, can be calculated.

The deformation is wrapped in the black and white stripe of the interferogram, and it is verified that the digital holographic interferometry can effectively measure the ultrasonic wavefield.

### 4.2. Phase Unwrapping in Measurement of 3D Ultrasonic Wavefields

The ultrasonic wavefields are obtained by phase unwrapping because the absolute value of the phase change is wrapped in the interferogram.

Fast two-dimensional phase-unwrapping algorithm based on sorting by reliability following a noncontinuous path (2D-SRNCP) [25] is used to process the unwrapping phase. The algorithm sorts by reliability, following a non-continuous path, and copes excellently with the noise that corrupts the real wrapped phase images.

**Figure 11:**
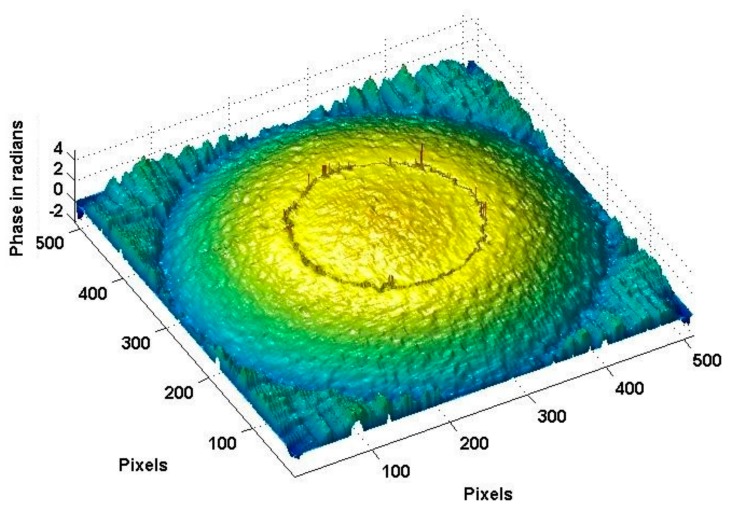
Phase image after unwrapping.

Figure 11 shows the true deformation of the phase in Figure 10 after using the 2D-SRNCP algorithm. As shown in Figure 11, some of the points after unwrapping are different from those of the initial model. Because the reconstructed object wave field is a speckle field, the amplitude and phase of the interferogram is subject to external constraints and perturbation. This random noise affects the quality of the image and the results of the unwrapping algorithm. The 2D-SRNCP algorithm is mainly based on sorting by reliability to solve the phase-wrapping. The error points in Figure 11 are mostly low reliability, and the noise more seriously affects the unwrapping algorithms of these points, so annular irregularities appear. The optimized unwrapping algorithm will be studied for error points in the next study.

**Figure 12:**
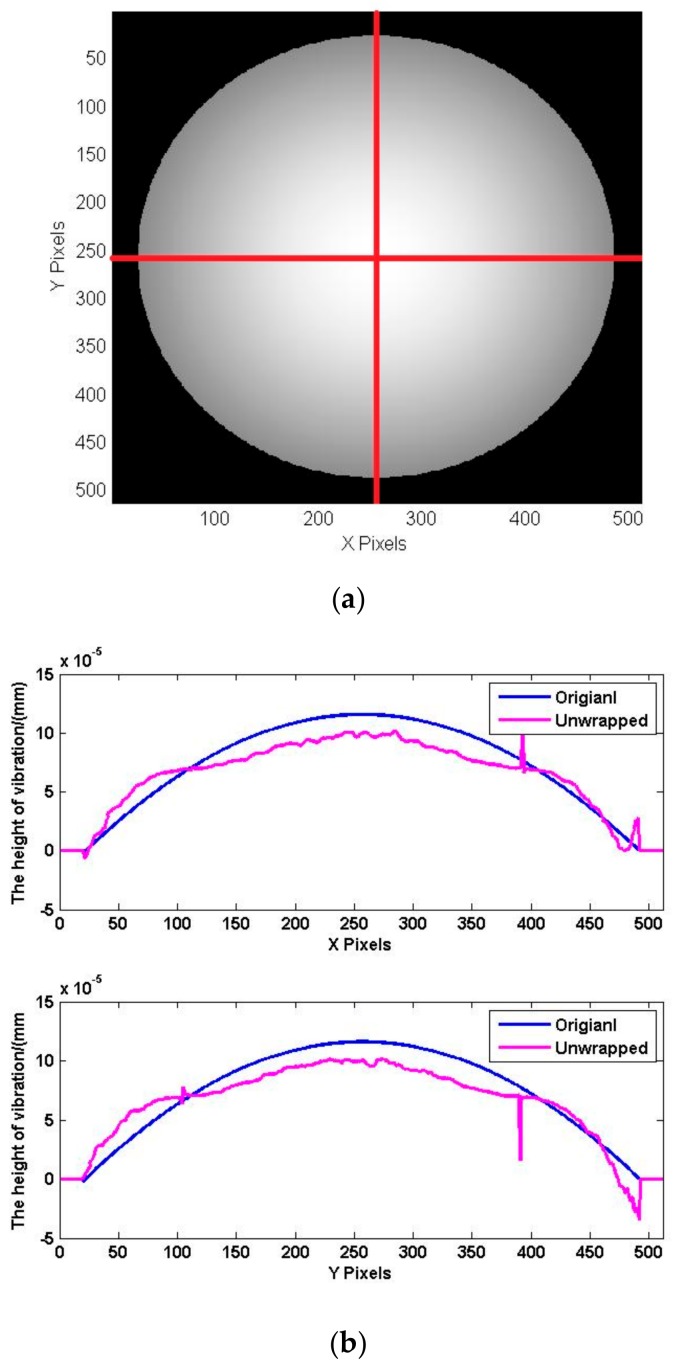
Phases of deformation. (**a**) Profile data; (**b**) data comparison.

To analyze the data more clearly, the one-dimensional profile data (X and Y directions) of the initial model and the unwrapped phases are obtained separately. As shown in Figure 12a, the profile data (red line) is selected in the image. The profile data are matched to the three-dimensional data, and the phase data of the profile data are obtained, as shown in Figure 12b. It can be confirmed again that the phase after unwrapping conforms to the initial model of vibration deformation. Comparing the two sets of curves shows that the greatest error is near the center of the circle. The maximum error is 0.28 μm, about 18%.

There is also a case when the phase is not wrapped at all, that is, when the height of the deformation is close to several or several tens of nanometers, there is no need for unwrapping.

## 5. Experimental Results

**Figure 13:**
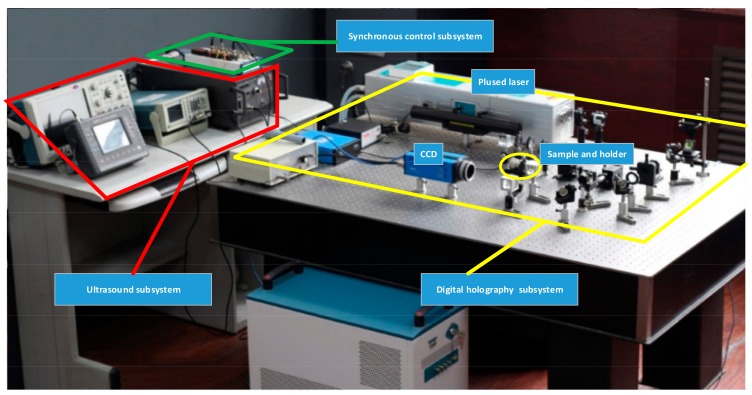
The proposed 3D Ultrasonic Wavefields measurement system.

Figure 13 shows the proposed 3D Ultrasonic Wavefields measurement system. The subsystem of digital holography consists of the CCD, the pulsed laser, and some optical components, which form the off-axis digital holographic optical path in Figure 4. The ultrasound subsystem consists of arbitrary waveform generator, power amplifier and ultrasonic transducer.

A preliminary experiment was carried out to verify the designed system. A dynamic ultrasonic wavefield generated by a piezoelectric ceramic sheet was measured using the designed system. A fixed piezoelectric ceramic sheet with a diameter of 25 mm, thickness of 0.2 mm, and frequency of 2700 Hz is used. The CCD pixel size is Δx×Δy=7.4 µm×7.4 µm, and it has a pixel resolution of 1200×1200. Because we used large specimens, we used a lens with an f=80 mm focal length instead of a high-power microscope to reduce the large spot size to fit the CCD. The distance from the sample to the CCD is 400 mm, the distance from the microscope to the image is 100 mm, and the imaging reduction ratio is 4. The frequency of the detected vibration is 2700 Hz, the period of the vibration is 370,000 ns, and the 8 ns pulse width of the pulsed laser is much less than 370,000 ns, therefore it is transient.

**Figure 14:**
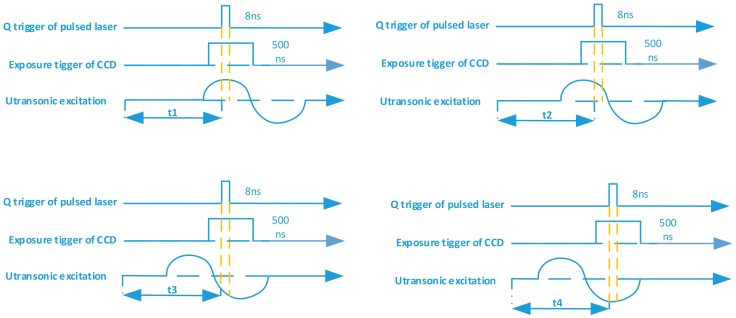
Synchronous control timing diagram in the experiment.

As shown in Figure 14, the dynamic ultrasonic wavefields at four different time instants are measured in this preliminary study. Four different time delays (*t*1, *t*2, *t*3, *t*4) are set up to obtain different ultrasonic wavefields.

**Figure 15:**
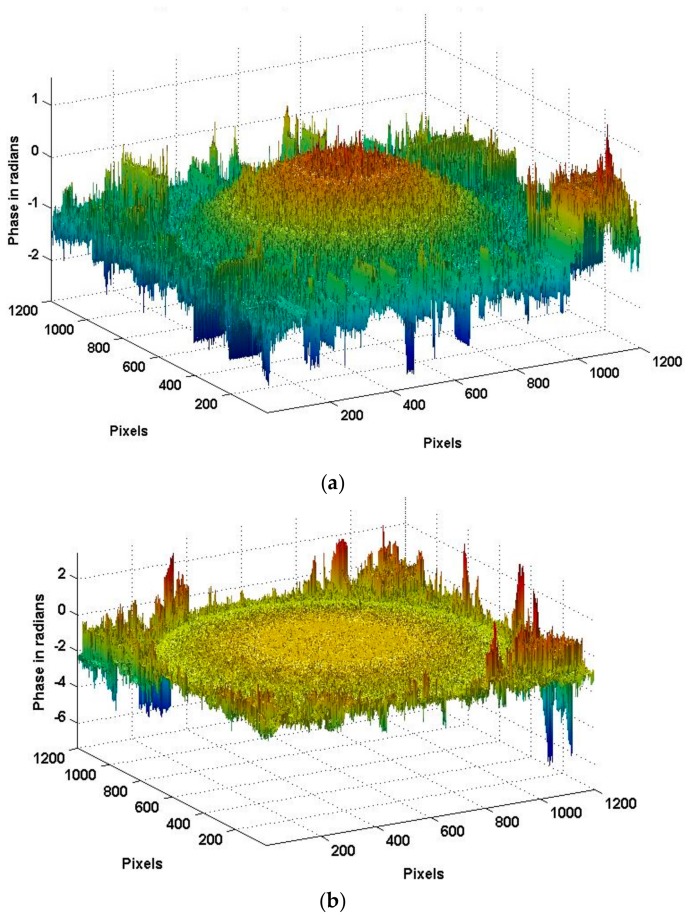
The surface topography at *t*1 and *t*2 moment. (**a**) Maximum surface topography at *t*1 moment; (**b**) the surface topography at *t*2 moment.

**Figure 16:**
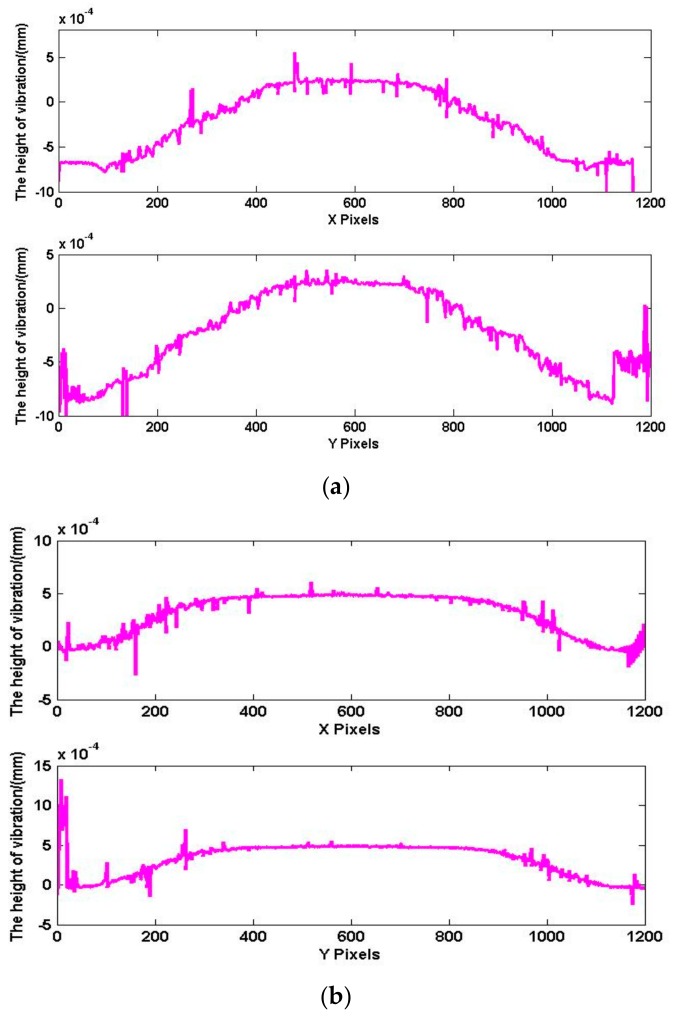
Data comparison at *t*1 and *t*2 moment. (**a**) Maximum surface topography at *t*1 moment; (**b**) the surface topography at *t*2 moment.

Figure 15 shows the wavefield at the transducer surface when the transducer is vibrating to the *t*1 moment and *t*2 moment, and t1 moment has the positive maximum amplitude. Due to the serious interference caused by stray light in the experiment, we used an initial median filter to mitigate interference from noise. As shown in Figure 12a, the cross-section (X direction and Y direction) is selected from the phase shown in Figure 16, and the results are shown in Figure 15. As shown in Figure 16a, the maximum amplitude is 0.89 μm. As shown in Figure 16b, the amplitude at t2 moment is 0.48 μm.

**Figure 17:**
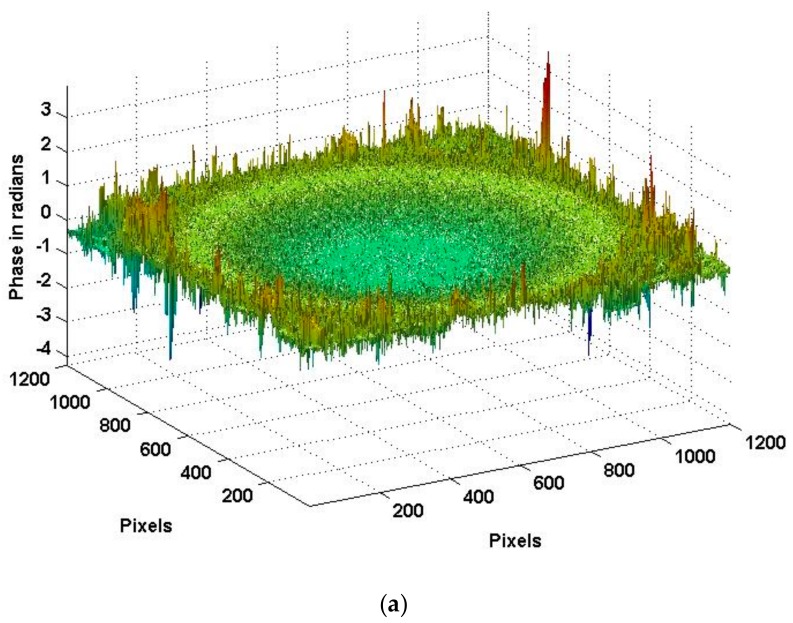

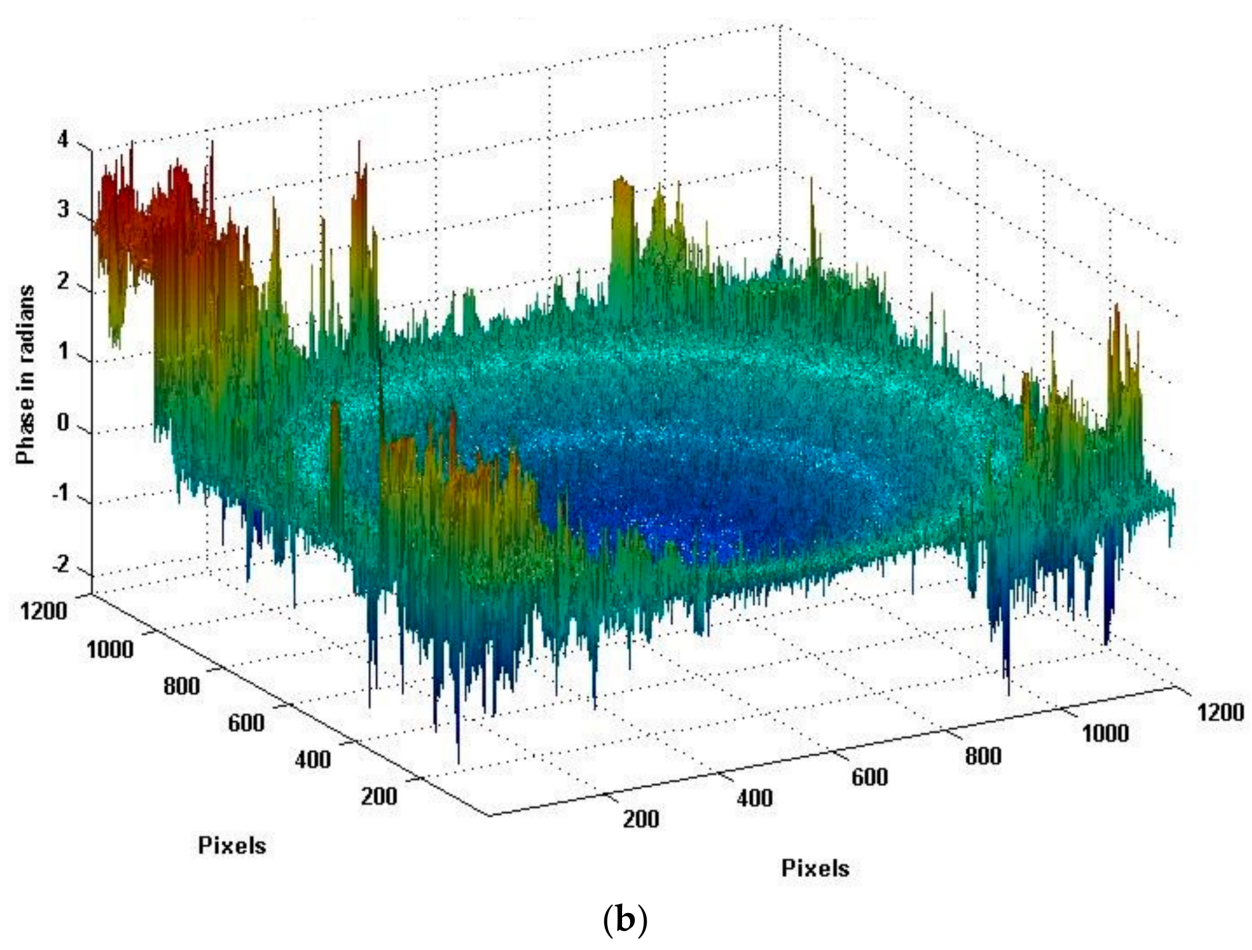
The surface topography at *t*3 and *t*4 moment. (**a**) The surface topography at *t*3 moment; (**b**) maximum surface topography at *t*4 moment.

**Figure 18:**
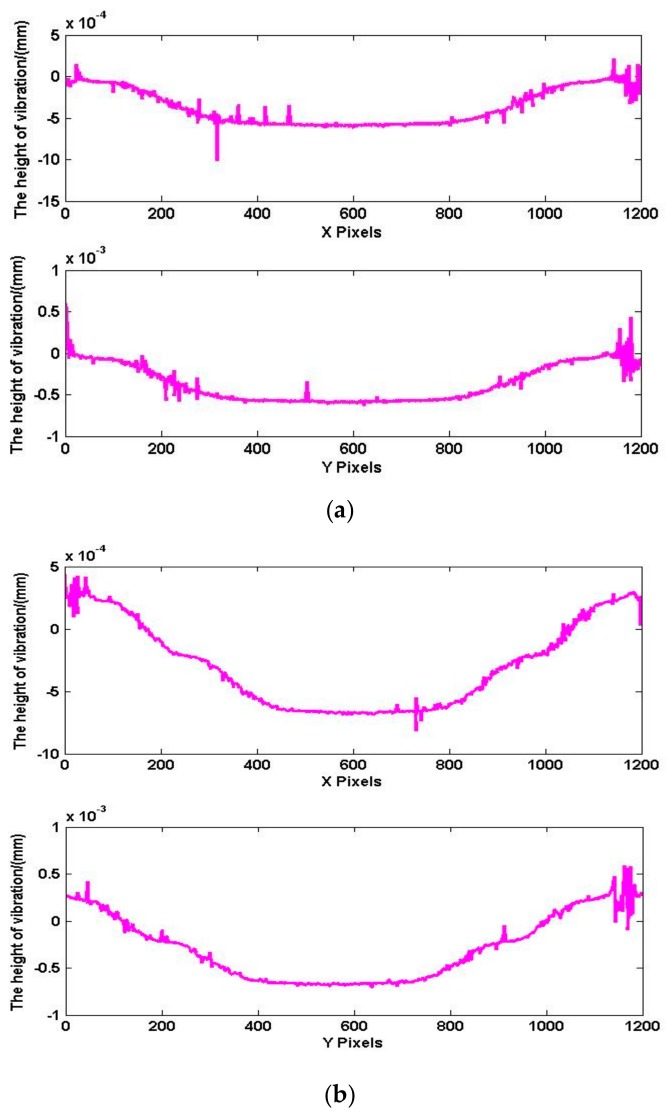
Data comparison at *t*3 and *t*4 moment. (**a**) The surface topography at *t*3 moment; (**b**) maximum surface topography at *t*4 moment.

Figure 17 and Figure 18 show the surface topography when the amplitude in the reverse direction. The process of data processing is the same as *t*1 and *t*2 moment. Figure 17a is the surface topography at t3 moment, and Figure 17b is the surface topography at *t*4 moment. The cross-section (X direction and Y direction) data are shown in Figure 18. The amplitude is 0.59 μm at *t*3 moment, and the maximum amplitude is 0.91 μm at t4 moment.

In order to verify the measurement data, the traditional time-averaged method is used to measure the same piezoelectric ceramic sheet. Because the frequency of the ultrasonic wavefields in the preliminary experiment is low, the vibration of the piezoelectric ceramic sheet could be measured by the time-averaged method. The optical subsystem in the designed system is used, and the pulsed laser is replaced by a continuous laser. Under the same experimental parameters, the amplitude of the vibration obtained by the time-averaged method is 0.75 μm. The time-averaged method measures the average of the vibration of the ultrasonic wavefields, and the method proposed in this paper measures the amplitude of the transient ultrasonic wavefields.

## 6. Conclusions

In this paper, the optical detection techniques act as the ultrasonic receiving array for ultrasonic imaging in order to overcome the challenging issues faced by the current ultrasonic transducer arrays. The method based on holographic interferometry is proposed to measure the dynamic ultrasonic wavefields, and the pulsed digital holographic microscopy system is designed. The consecutive sequence of interferograms of ultrasonic wavefields are calculated from the holograms, which are recorded at different time sequences by the system. Phase unwrapping is used to recover the deformation distribution of transient wavefields from the interferograms. Computer simulation verified the feasibility of the proposed measurement method. In the experiment, the pulsed digital holographic microscopy system has been used to capture and measure dynamic ultrasonic wavefields generated by a piezoelectric ceramic sheet. The experimental results also verified the feasibility of the proposed method.
